# Crystal structure and Hirshfeld surface analysis of ethyl 2-amino-4-(4-chloro­phen­yl)-5,6,7,8,9,10-hexa­hydro­cyclo­octa­[*b*]pyridine-3-carboxyl­ate

**DOI:** 10.1107/S2056989026003087

**Published:** 2026-03-27

**Authors:** Srinivasan Pazhamalai, Chandiran Jayakodi, Velayutham Mahalakshmi, Rajendran Arivu Selvan, Sivashanmugam Selvanayagam

**Affiliations:** a Department of Chemistry Annamalai University, Annamalainagar Chidambaram 608 002 India; b PG & Research Department of Physics, Government Arts College, Melur 625 106, India; Vienna University of Technology, Austria

**Keywords:** pyridine derivative, inter­molecular hydrogen bonds, Hirshfeld surface analysis, crystal structure.

## Abstract

In the title compound, C_20_H_23_ClN_2_O_2_, the cyclo­octene ring has a boat–chair conformation. The ethyl formate moiety is equally disordered over two positions. The dihedral angle between the pyridine and chloro­phenyl rings is 81.33 (13)°. An intra­molecular N—H⋯O hydrogen bond helps to stabilize the mol­ecular conformation. In the crystal, N—H⋯N hydrogen-bonding leads to the formation of dimers with an *R*_2_^2^(8) graph-set motif. Additional consolidation of the packing in the crystal is achieved through C—H⋯π and π–π stacking inter­actions. The inter­molecular inter­actions were qu­anti­fied and analysed using Hirshfeld surface analysis, revealing that H⋯H inter­actions contribute by far the most to the crystal packing (63.6%).

## Abstract 2

In the title compound, the cyclo­octene ring has a boat–chair conformation.

## 1. Chemical context

The synthesis of functionalized N-heterocycles is an imprtant objective in organic and medicinal chemistry, as these moieties constitute the core of over 60% of US Food and Drug Administration (FDA) approved small-mol­ecule drugs. Among these, the pyridine entity is arguably the most ubiquitous, appearing in essential vitamins such as nicotinic acid and various synthetic pharmaceuticals like Delafloxacin (Van Bambeke, 2015 ▸). Within this family, 2-amino­nicotinate esters have garnered significant attention as privileged scaffolds. These multifunctional mol­ecules possess a unique 1,2,3-arrangement of substituents – an amino group and a carboxyl­ate ester – which provides a rich landscape for both mol­ecular recognition and chemical transformation (Bagley *et al.*, 2015 ▸). The chemical appeal of 2-amino­nicotinate esters lies in their synthetic versatility. They serve as precursors for the construction of fused heterocyclic systems, such as pyrido[2,3-*d*]pyrimidines and 1,8-naphthyridines, which are themselves pharmacologically active. Modern synthetic routes have shifted toward multicomponent reactions (MCRs), which allow for the one-pot assembly of these esters from readily available aldehydes, malono­nitriles, and alcohols. These methods are beneficial for their economy and compliance with green chemistry principles, often utilizing heterogeneous catalysts or aqueous media (Shaaban *et al.*, 2020 ▸). Biologically, these esters are highly multifunctional. The amino and ester groups provide critical hydrogen-bonding sites that facilitate high-affinity binding to various enzyme pockets. It has been demonstrated that deriv­atives of 2-amino­nicotinates exhibit a broad spectrum of bio-activities, including anti-inflammatory and analgesic when acting as non-selective or COX-2 selective inhibitors (Bekhit *et al.*, 2017 ▸), and anti­microbial by demonstrating potency against Gram-positive and Gram-negative pathogens through disrupting metabolic pathways (El-Gazzar & Hafez, 2021 ▸). Given the rising challenge of drug resistance in both oncology and infectious diseases, the development of diverse 2-amino­nicotinate derivatives offers a promising avenue for the discovery of next-generation therapeutic agents.
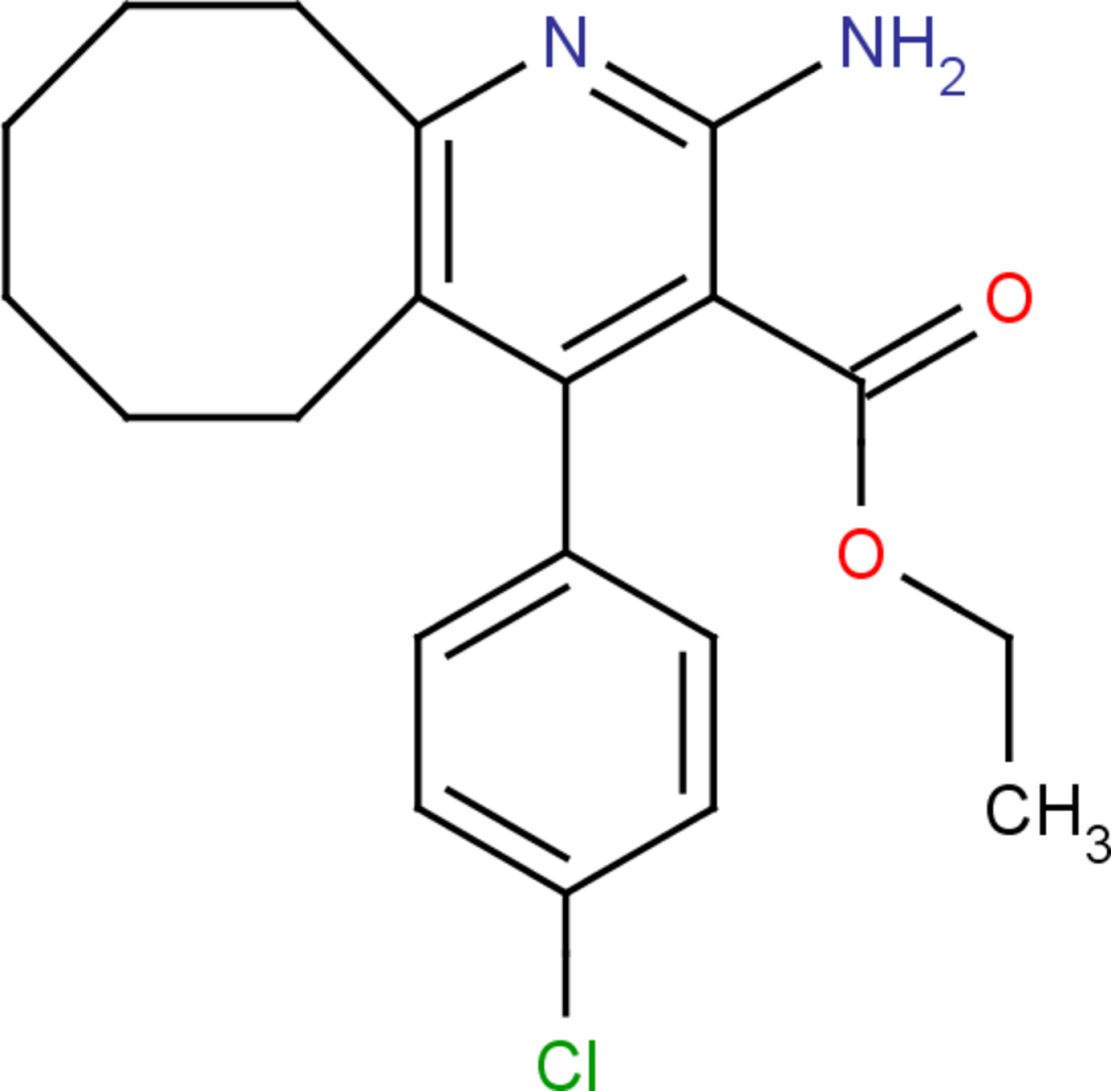


In this work, we describe the synthesis, structure and Hirshfeld surface analysis of the title compound, C_20_H_23_ClN_2_O_2_, (I).

## 2. Structural commentary

**Figure 1:**
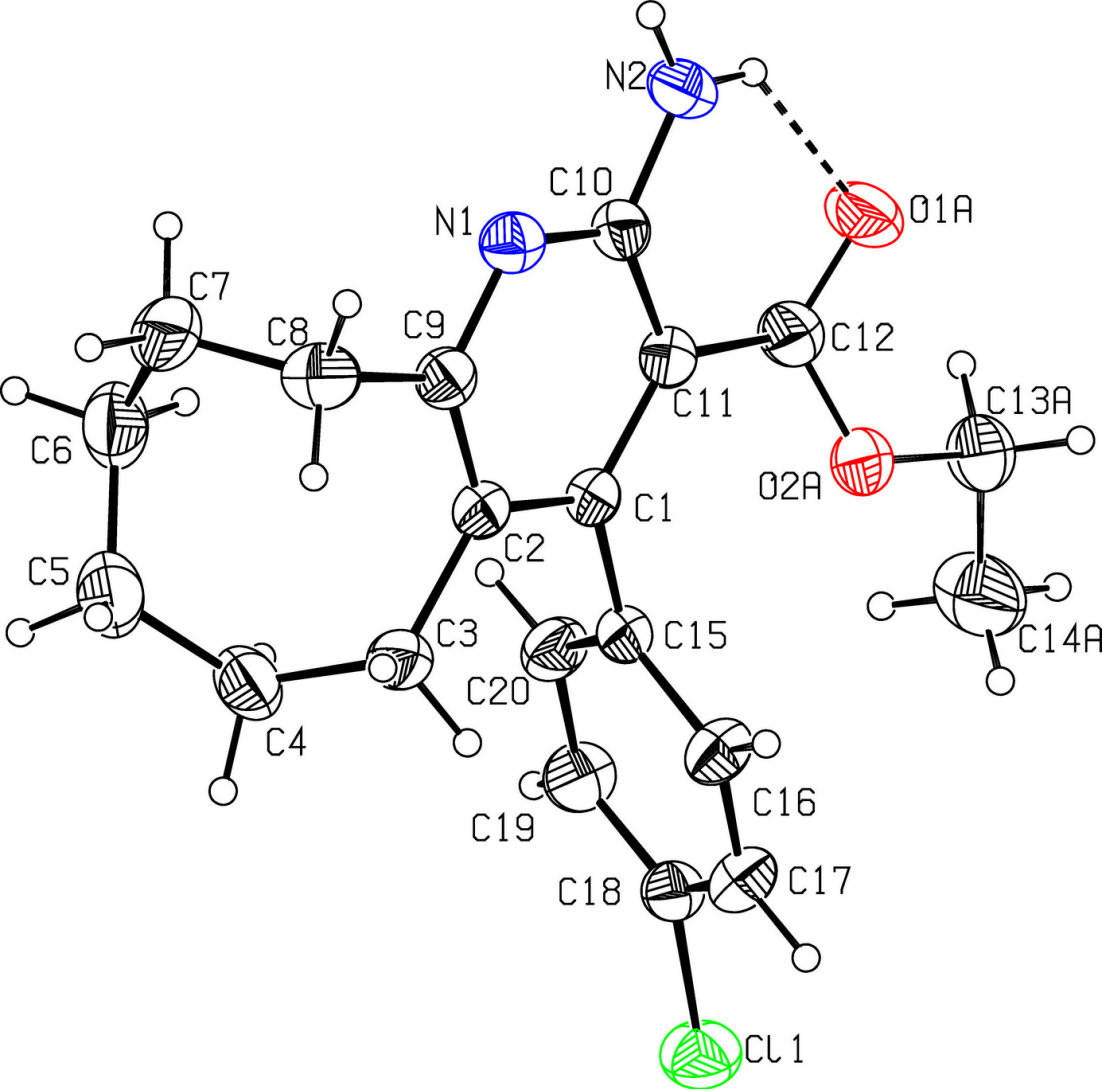
A view of the mol­ecular structure of compound (I), showing the atom labelling. Displacement ellipsoids are drawn at the 30% probability level. Only one of the disordered parts of the ethyl formate chain is displayed for clarity; the intra­molecular hydrogen bond is shown as a dashed line.

The mol­ecular structure of (I) is displayed in Fig. 1 ▸. The pyridine ring (C1/C2/C9/N1/C10/C11) is essentially planar, with a maximum deviation of −0.016 (3) Å for atom C11, while its attached amino nitro­gen atom N2 deviates by 0.005 (3) Å from this plane. The chloro­phenyl ring is also almost planar, and its attached chlorine atom deviates by −0.031 (1) Å from this plane. The dihedral angle between the pyridine and chloro­phenyl rings is 81.33 (13)°. The cyclo­octene ring (C2–C9) has a boat–chair conformation based on a puckering analysis and endocyclic torsion angles (Evans & Boeyens, 1988 ▸). An intra­molecular N—H⋯O hydrogen bond (Table 1 ▸) between atoms N2 and O1*A* contributes to the stability of the mol­ecular conformation. This N2—H2*B*⋯O1*A* inter­action generates an *S*(6) ring motif (Bernstein *et al.*, 1995 ▸), as shown in Fig. 1 ▸.

## 3. Supra­molecular features

**Figure 2:**
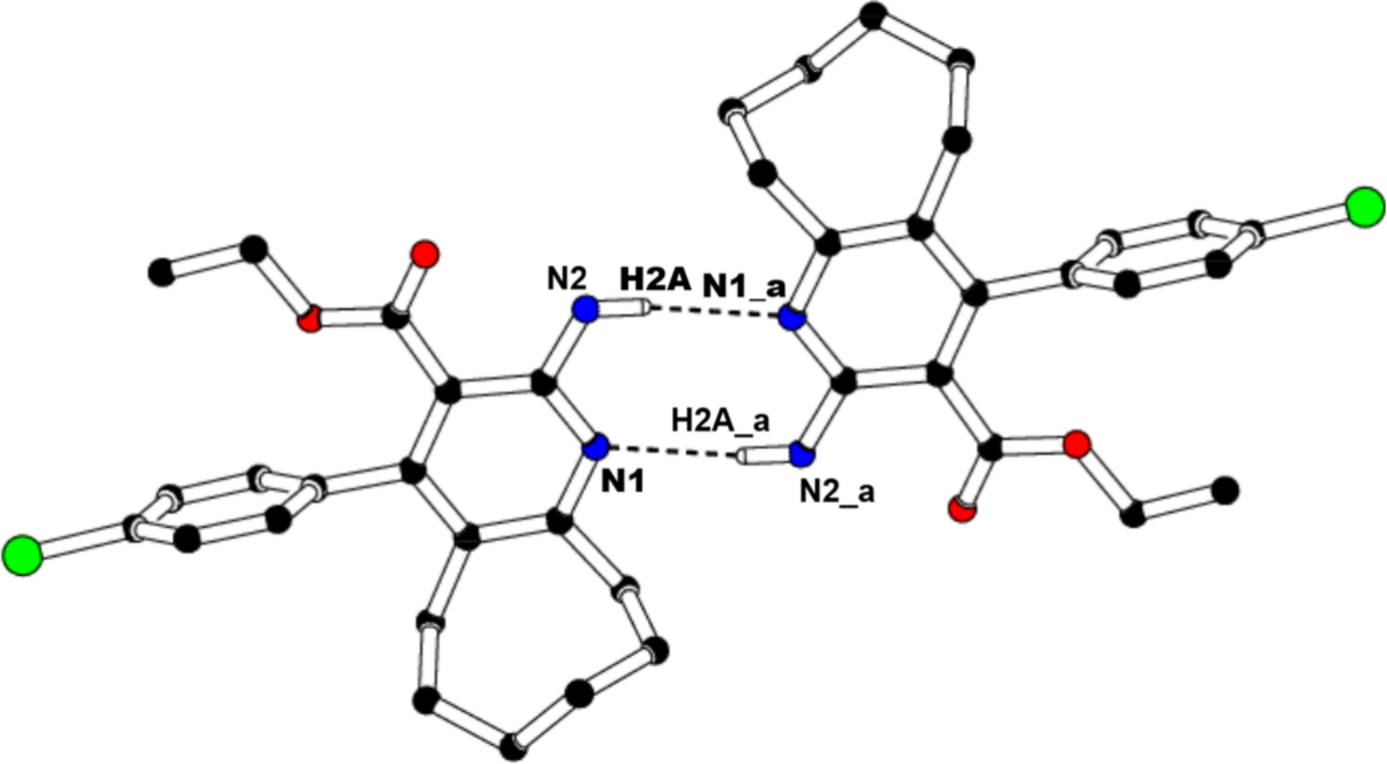
The formation of a centrosymmetric dimer in the crystal structure of (I) through N—H⋯N hydrogen bonds. For clarity, H atoms not involved in these inter­actions have been omitted, and only one of the disordered parts of the ethyl formate chain is displayed. [Symmetry code: (*a*) −*x*, −*y* − 1, −*z* + 1].

**Figure 3:**
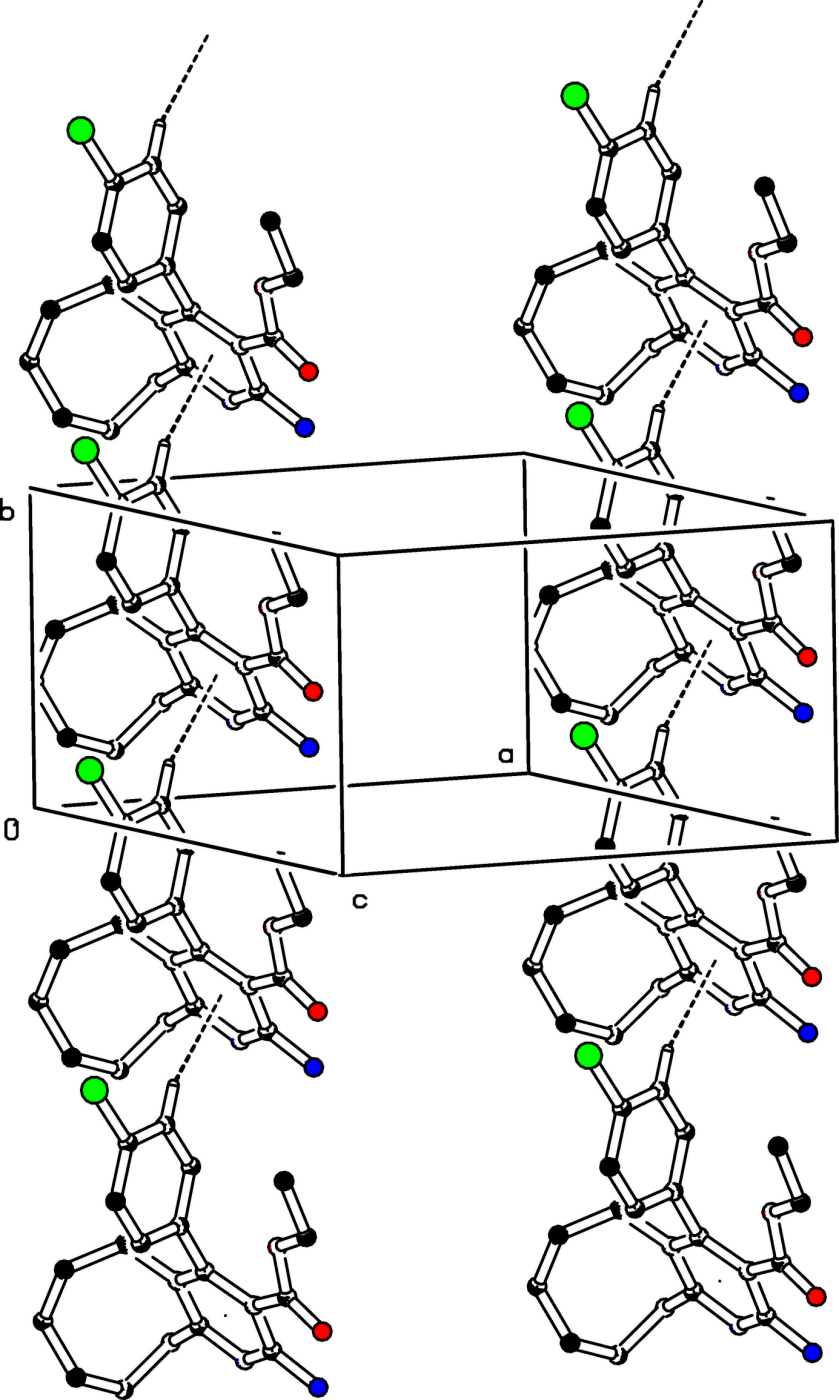
The crystal packing of (I). C—H⋯π inter­actions are shown as dashed lines. For clarity, H atoms not involved in these inter­actions have been omitted, and only one of the disordered parts of the ethyl formate chain is displayed.

In the crystal, mol­ecules associate pairwise through N2—H2*A*⋯N1^i^ hydrogen bonds (Table 1 ▸) into inversion dimers with an 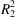
(8) graph-set motif (Etter *et al.*, 1990 ▸; Bernstein *et al.*, 1995 ▸), as shown in Fig. 2 ▸. Mol­ecules are linked into chains parallel to [010] by C—H⋯π inter­actions, C17—H17⋯*Cg*, where *Cg* is the centroid of the pyridine ring (Table 1 ▸, Fig. 3 ▸). Moreover, π–π inter­actions are observed between the centroids of inversion-related pyridine rings with a centroid-to-centroid distance of 3.764 (2) Å and a slippage of 0.711 Å.

## 4. Hirshfeld surface analysis

**Figure 4:**
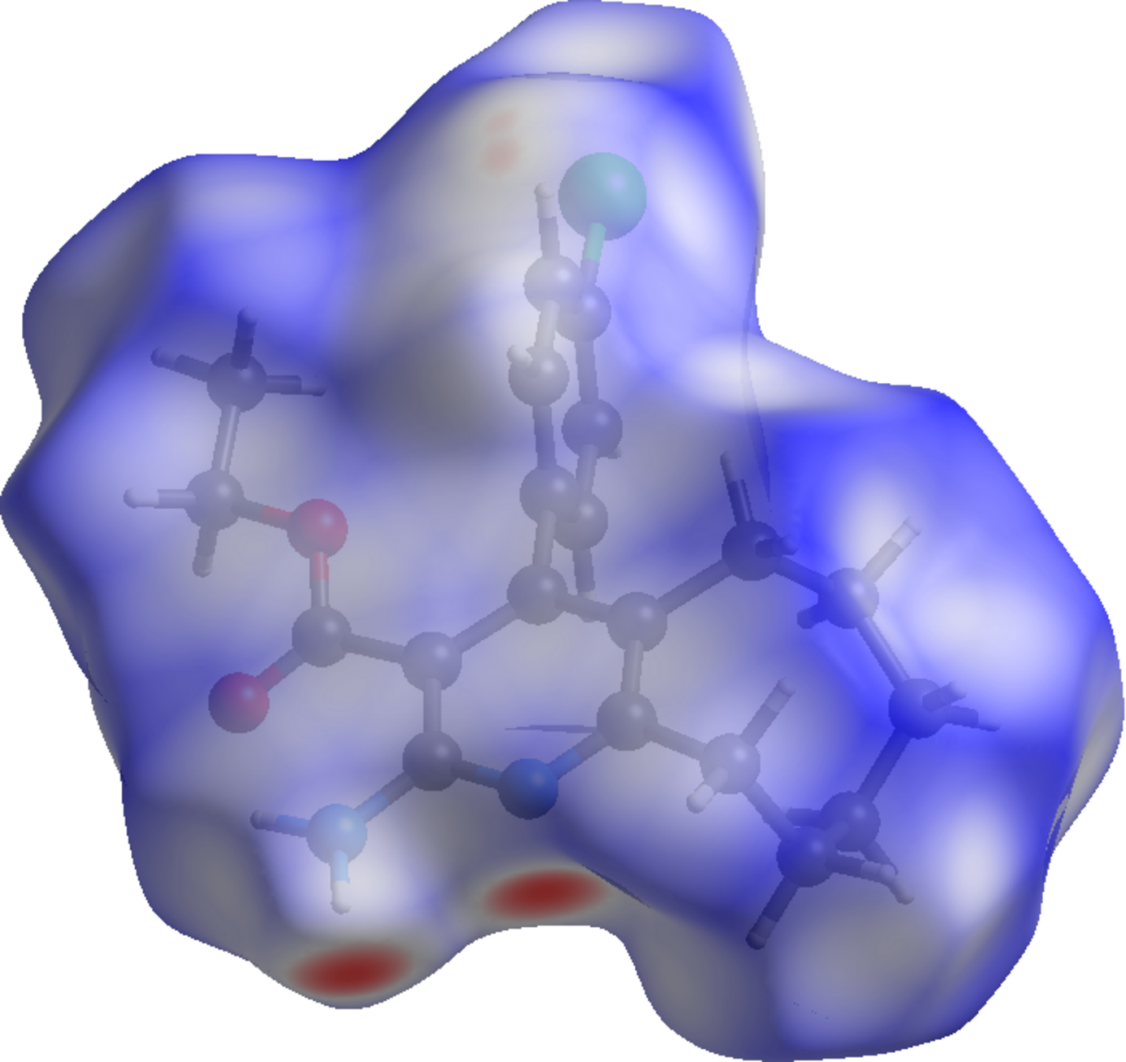
A view of the Hirshfeld surface mapped over *d*_norm_ for compound (I).

**Figure 5:**
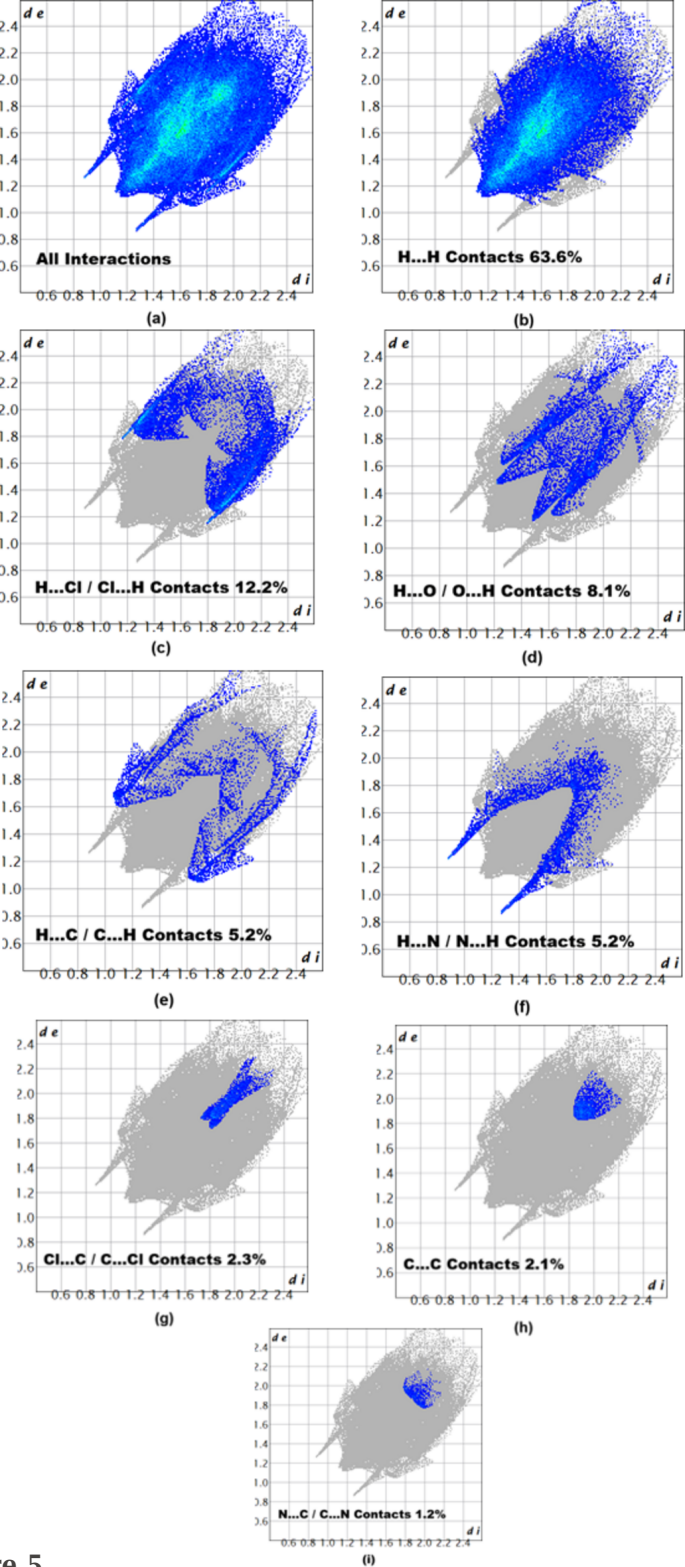
Two-dimensional fingerprint plots for the compound (I), showing (*a*) all inter­actions, and delineated into (*b*) H⋯H, (*c*) H⋯Cl/Cl⋯H, (*d*) H⋯O/O⋯H, (*e*) H⋯C/C⋯H, (*f*) H⋯N/N⋯H, (*g*) Cl⋯C/C⋯Cl, (*h*) C⋯C and (i) N⋯C/C⋯N inter­actions. The *d*_i_ and *d*_e_ values are the closest inter­nal and external distances (in Å) from given points on the Hirshfeld surface.

A Hirshfeld surface (HS) analysis (Spackman & Jayatilaka, 2009 ▸) was carried out using *CrystalExplorer* (Spackman *et al.*, 2021 ▸) to characterize and qu­antify the inter­molecular inter­actions in the title compound. For this purpose, a model without the disorder of the ethyl formate moiety was used (the ethyl formate moiety was set to full occupancy for the Hirshfeld surface analysis). The HS mapped over *d*_norm_ is illustrated in Fig. 4 ▸, where deep-red spots indicative of strong inter­actions occur at N1 and H2*A*, and these atoms are responsible for the inter­molecular hydrogen bonds discussed above. The associated two-dimensional fingerprint plots (McKinnon *et al.*, 2007 ▸) provide qu­anti­tative information about the non-covalent inter­actions in the crystal packing in terms of the percentage contribution of the inter­atomic contacts (Spackman & McKinnon, 2002 ▸). As shown in Fig. 5 ▸, the overall two-dimensional fingerprint plot for compound (I) is delineated into H⋯H, H⋯Cl/Cl⋯H, H⋯O/O⋯H, H⋯C/ C⋯H, H⋯N/N⋯H, Cl⋯C/C⋯Cl, C⋯C and N⋯C/C⋯N contacts, revealing that H⋯H inter­actions are by far the main contributor to the crystal packing.

## 5. Synthesis and crystallization

Compound (I) was prepared using a mixture of cyclo­octa­none (1.0 mmol, 0.126 g), 4-chloro­benzaldehyde (1.0 mmol, 0.140 g), ethyl cyano­acetate (1.0 mmol, 0.113 g) and ammonium acetate (1.5 mmol, 0.116 g) taken in a 100 ml round-bottom flask and dissolved using ethanol. The resulting solution was heated under reflux with stirring for 4–6 h. The progress of the reaction was periodically monitored by thin-layer chromatography using ethyl acetate:hexane (3:7) as the eluent. Upon completion of the reaction, the mixture was allowed to cool to room temperature, leading to the formation of a solid precipitate. The solid was collected by vacuum filtration and washed with cold ethanol to remove residual impurities. For final purification, the product was recrystallized from ethanol solution, yielding clean, well-formed crystals suitable for single crystal X-ray diffraction studies.

## 6. Refinement

Crystal data, data collection and structure refinement details are summarized in Table 2 ▸. Hydrogen atoms were placed in idealized positions and allowed to ride on their parent atoms, N—H = 0.86 Å and C—H = 0.93–0.97 Å, with *U*_iso_(H) = 1.5*U*_eq_ for methyl H atoms and *U*_iso_(H) = 1.2*U*_eq_(C,N) for all other H atoms. The ethyl formate group (C12, O1, O2, C13, C14) is equally disordered over two sets of sites. For modelling of this disorder, pairs of C—O and C=O bond lengths were restrained to 1.31 (1) and 1.20 (1) Å, respectively, and the displacement parameters of all atoms involved were restrained to be within 0.01 Å of each other.

## Supplementary Material

Crystal structure: contains datablock(s) I, shelx. DOI: 10.1107/S2056989026003087/wm5792sup1.cif

Structure factors: contains datablock(s) I. DOI: 10.1107/S2056989026003087/wm5792Isup2.hkl

Supporting information file. DOI: 10.1107/S2056989026003087/wm5792Isup3.cml

CCDC reference: 2539747

Additional supporting information:  crystallographic information; 3D view; checkCIF report

## Figures and Tables

**Table 1:** Hydrogen-bond geometry (Å, °) *Cg* is the centroid of pyridine ring (C1/C2/C9/N1/C10/C11).

*D*—H⋯*A*	*D*—H	H⋯*A*	*D*⋯*A*	*D*—H⋯*A*
N2—H2*B*⋯O1*A*	0.86	1.96	2.605 (10)	131
N2—H2*A*⋯N1^i^	0.86	2.29	3.129 (3)	165
C17—H17⋯*Cg*^ii^	0.93	2.84	3.657 (3)	147

Symmetry codes: (i) 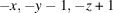; (ii) 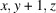.

**Table 2:** Experimental details

Crystal data	
Chemical formula	C_20_H_23_ClN_2_O_2_
*M*_r_	358.85
Crystal system, space group	Monoclinic, *P*2_1_/*n*
Temperature (K)	300
*a*, *b*, *c* (Å)	13.2386 (12), 7.2770 (7), 19.7223 (18)
β (°)	101.461 (3)
*V* (Å^3^)	1862.1 (3)
*Z*	4
Radiation type	Mo *K*α
μ (mm^−1^)	0.22
Crystal size (mm)	0.26 × 0.12 × 0.08

Data collection	
Diffractometer	Bruker APEXII CCD
Absorption correction	Multi-scan (*SADABS*; Krause *et al.*, 2015 ▸)
*T*_min_, *T*_max_	0.945, 0.983
No. of measured, independent and observed [*I* > 2σ(*I*)] reflections	34945, 4649, 2682
*R*_int_	0.050
(sin θ/λ)_max_ (Å^−1^)	0.669

Refinement	
*R*[*F*^2^ > 2σ(*F*^2^)], *wR*(*F*^2^), *S*	0.064, 0.207, 1.06
No. of reflections	4649
No. of parameters	264
No. of restraints	108
H-atom treatment	H-atom parameters constrained
Δρ_max_, Δρ_min_ (e Å^−3^)	0.55, −0.27

Computer programs: *APEX3* and *SAINT* (Bruker, 2017 ▸), *SHELXT* (Sheldrick, 2015*a* ▸), *SHELXL* (Sheldrick, 2015*b* ▸), *ORTEP-3 for Windows* (Farrugia, 2012 ▸), *PLATON* (Spek, 2020 ▸) and *publCIF* (Westrip, 2010 ▸).

## supplementary crystallographic information

### Ethyl 2-amino-4-(4-chlorophenyl)-5,6,7,8,9,10-hexahydrocycloocta[b]pyridine-3-carboxylate Crystal data

**Table:**

C_20_H_23_ClN_2_O_2_	*F*(000) = 760
*M**_r_* = 358.85	*D*_x_ = 1.280 Mg m^−^^3^
Monoclinic, *P*2_1_/*n*	Mo *K*α radiation, λ = 0.71073 Å
*a* = 13.2386 (12) Å	Cell parameters from 9916 reflections
*b* = 7.2770 (7) Å	θ = 3.0–26.3°
*c* = 19.7223 (18) Å	µ = 0.22 mm^−^^1^
β = 101.461 (3)°	*T* = 300 K
*V* = 1862.1 (3) Å^3^	Block, colourless
*Z* = 4	0.26 × 0.12 × 0.08 mm

### Ethyl 2-amino-4-(4-chlorophenyl)-5,6,7,8,9,10-hexahydrocycloocta[b]pyridine-3-carboxylate Data collection

**Table:**

Bruker APEXII CCD diffractometer	2682 reflections with *I* > 2σ(*I*)
Radiation source: i-mu-s microfocus source	*R*_int_ = 0.050
φ and ω scans	θ_max_ = 28.4°, θ_min_ = 2.1°
Absorption correction: multi-scan (SADABS; Krause *et al.*, 2015)	*h* = −17→17
*T*_min_ = 0.945, *T*_max_ = 0.983	*k* = −9→9
34945 measured reflections	*l* = −26→26
4649 independent reflections	

### Ethyl 2-amino-4-(4-chlorophenyl)-5,6,7,8,9,10-hexahydrocycloocta[b]pyridine-3-carboxylate Refinement

**Table:**

Refinement on *F*^2^	Hydrogen site location: inferred from neighbouring sites
Least-squares matrix: full	H-atom parameters constrained
*R*[*F*^2^ > 2σ(*F*^2^)] = 0.064	*w* = 1/[σ^2^(*F*_o_^2^) + (0.0769*P*)^2^ + 1.3578*P*] where *P* = (*F*_o_^2^ + 2*F*_c_^2^)/3
*wR*(*F*^2^) = 0.207	(Δ/σ)_max_ < 0.001
*S* = 1.06	Δρ_max_ = 0.55 e Å^−^^3^
4649 reflections	Δρ_min_ = −0.27 e Å^−^^3^
264 parameters	Extinction correction: SHELXL (Sheldrick, 2015b), Fc^*^=kFc[1+0.001xFc^2^λ^3^/sin(2θ)]^-1/4^
108 restraints	Extinction coefficient: 0.0053 (14)

### Ethyl 2-amino-4-(4-chlorophenyl)-5,6,7,8,9,10-hexahydrocycloocta[b]pyridine-3-carboxylate Special details

**Table:**

Geometry. All esds (except the esd in the dihedral angle between two l.s. planes) are estimated using the full covariance matrix. The cell esds are taken into account individually in the estimation of esds in distances, angles and torsion angles; correlations between esds in cell parameters are only used when they are defined by crystal symmetry. An approximate (isotropic) treatment of cell esds is used for estimating esds involving l.s. planes.

### Ethyl 2-amino-4-(4-chlorophenyl)-5,6,7,8,9,10-hexahydrocycloocta[b]pyridine-3-carboxylate Fractional atomic coordinates and isotropic or equivalent isotropic displacement parameters (Å^2^)

**Table:**

	*x*	*y*	*z*	*U*_iso_*/*U*_eq_	Occ. (<1)
N1	0.07369 (17)	−0.2785 (3)	0.52707 (12)	0.0531 (6)	
N2	−0.00533 (19)	−0.3486 (3)	0.41712 (13)	0.0654 (7)	
H2A	−0.034366	−0.439014	0.433851	0.078*	
H2B	−0.017722	−0.329464	0.373218	0.078*	
Cl1	0.44196 (7)	0.62949 (12)	0.41161 (5)	0.0778 (3)	
C1	0.17789 (18)	0.0195 (3)	0.48544 (13)	0.0458 (6)	
C2	0.19016 (19)	−0.0236 (3)	0.55599 (13)	0.0485 (6)	
C3	0.2612 (2)	0.0864 (4)	0.61089 (15)	0.0604 (7)	
H3A	0.229762	0.094328	0.651235	0.072*	
H3B	0.265523	0.210315	0.593602	0.072*	
C4	0.3700 (2)	0.0133 (5)	0.63391 (17)	0.0750 (9)	
H4A	0.396761	−0.014633	0.592727	0.090*	
H4B	0.412006	0.111600	0.657960	0.090*	
C6	0.3400 (3)	−0.3296 (6)	0.6488 (2)	0.0872 (11)	
H6A	0.388293	−0.428382	0.664182	0.105*	
H6B	0.332028	−0.320339	0.598970	0.105*	
C8	0.1485 (3)	−0.2405 (4)	0.64719 (15)	0.0675 (8)	
H8A	0.084039	−0.293594	0.653997	0.081*	
H8B	0.163888	−0.136091	0.678107	0.081*	
C7	0.2338 (3)	−0.3826 (5)	0.66593 (18)	0.0817 (10)	
H7A	0.210848	−0.496052	0.641912	0.098*	
H7B	0.243729	−0.407132	0.715151	0.098*	
C5	0.3855 (3)	−0.1551 (5)	0.6802 (2)	0.0858 (11)	
H5A	0.458980	−0.173647	0.695896	0.103*	
H5B	0.356562	−0.129580	0.720655	0.103*	
C9	0.1357 (2)	−0.1747 (4)	0.57360 (14)	0.0499 (6)	
C10	0.05992 (19)	−0.2364 (4)	0.45992 (14)	0.0508 (6)	
C11	0.1101 (2)	−0.0837 (4)	0.43593 (13)	0.0501 (6)	
C15	0.24158 (19)	0.1722 (3)	0.46504 (13)	0.0471 (6)	
C16	0.2137 (2)	0.3543 (4)	0.46938 (16)	0.0581 (7)	
H16	0.152775	0.382814	0.483821	0.070*	
C17	0.2744 (2)	0.4943 (4)	0.45269 (15)	0.0605 (7)	
H17	0.254742	0.616217	0.455904	0.073*	
C18	0.3638 (2)	0.4525 (4)	0.43137 (14)	0.0548 (7)	
C19	0.3934 (2)	0.2739 (4)	0.42640 (17)	0.0650 (8)	
H19	0.454033	0.246819	0.411419	0.078*	
C20	0.3327 (2)	0.1336 (4)	0.44375 (16)	0.0594 (7)	
H20	0.353465	0.012167	0.441067	0.071*	
C12	0.0893 (2)	−0.0450 (4)	0.36086 (15)	0.0642 (8)	
O1A	0.0236 (10)	−0.1269 (16)	0.3190 (5)	0.085 (3)	0.51 (2)
O1B	0.0739 (13)	−0.1670 (13)	0.3166 (5)	0.088 (3)	0.49 (2)
O2A	0.1083 (12)	0.1269 (14)	0.3464 (11)	0.063 (2)	0.51 (2)
O2B	0.1305 (15)	0.1061 (19)	0.3421 (12)	0.081 (4)	0.49 (2)
C13A	0.0965 (17)	0.178 (3)	0.2723 (15)	0.075 (3)	0.51 (2)
H13A	0.104132	0.068990	0.245334	0.091*	0.51 (2)
H13B	0.028133	0.227566	0.255673	0.091*	0.51 (2)
C13B	0.1142 (19)	0.148 (3)	0.2708 (16)	0.080 (3)	0.49 (2)
H13C	0.044942	0.114708	0.247579	0.095*	0.49 (2)
H13D	0.163390	0.083931	0.248771	0.095*	0.49 (2)
C14A	0.1750 (15)	0.316 (3)	0.2633 (8)	0.112 (4)	0.51 (2)
H14A	0.166505	0.347352	0.215229	0.134*	0.51 (2)
H14B	0.166787	0.423928	0.289563	0.134*	0.51 (2)
H14C	0.242641	0.265654	0.279244	0.134*	0.51 (2)
C14B	0.1308 (13)	0.3638 (19)	0.2693 (8)	0.085 (3)	0.49 (2)
H14D	0.121116	0.404984	0.222240	0.103*	0.49 (2)
H14E	0.081895	0.423836	0.291869	0.103*	0.49 (2)
H14F	0.199453	0.393290	0.293052	0.103*	0.49 (2)

### Ethyl 2-amino-4-(4-chlorophenyl)-5,6,7,8,9,10-hexahydrocycloocta[b]pyridine-3-carboxylate Atomic displacement parameters (Å^2^)

**Table:**

	*U*^11^	*U*^22^	*U*^33^	*U*^12^	*U*^13^	*U*^23^
N1	0.0523 (12)	0.0505 (13)	0.0574 (14)	−0.0021 (10)	0.0134 (10)	−0.0020 (11)
N2	0.0636 (15)	0.0623 (15)	0.0640 (15)	−0.0174 (12)	−0.0026 (12)	−0.0024 (12)
Cl1	0.0826 (6)	0.0708 (5)	0.0807 (6)	−0.0202 (4)	0.0181 (4)	0.0063 (4)
C1	0.0449 (13)	0.0400 (13)	0.0505 (14)	0.0051 (10)	0.0044 (10)	−0.0004 (11)
C2	0.0524 (14)	0.0402 (13)	0.0507 (15)	0.0039 (11)	0.0053 (11)	−0.0012 (11)
C3	0.0747 (19)	0.0500 (16)	0.0521 (15)	−0.0059 (14)	0.0022 (14)	−0.0014 (12)
C4	0.0681 (19)	0.087 (2)	0.0620 (18)	−0.0129 (18)	−0.0056 (15)	0.0080 (17)
C6	0.083 (2)	0.088 (3)	0.082 (2)	0.026 (2)	−0.0030 (19)	0.001 (2)
C8	0.084 (2)	0.0660 (19)	0.0550 (17)	−0.0136 (16)	0.0210 (15)	−0.0035 (15)
C7	0.109 (3)	0.064 (2)	0.066 (2)	−0.0059 (19)	0.0022 (19)	0.0197 (16)
C5	0.072 (2)	0.097 (3)	0.081 (2)	0.006 (2)	−0.0015 (18)	0.016 (2)
C9	0.0498 (14)	0.0486 (14)	0.0520 (15)	0.0039 (11)	0.0115 (11)	−0.0022 (12)
C10	0.0449 (13)	0.0492 (15)	0.0558 (16)	0.0026 (11)	0.0038 (11)	−0.0024 (12)
C11	0.0517 (14)	0.0471 (14)	0.0491 (14)	0.0035 (12)	0.0044 (11)	0.0013 (12)
C15	0.0497 (14)	0.0438 (13)	0.0446 (13)	0.0015 (11)	0.0019 (10)	−0.0013 (11)
C16	0.0605 (16)	0.0469 (15)	0.0703 (19)	0.0066 (12)	0.0210 (14)	0.0046 (13)
C17	0.0732 (19)	0.0438 (14)	0.0666 (18)	0.0060 (13)	0.0186 (15)	0.0027 (13)
C18	0.0607 (16)	0.0505 (15)	0.0507 (15)	−0.0059 (13)	0.0050 (12)	0.0021 (12)
C19	0.0569 (16)	0.0631 (19)	0.078 (2)	0.0010 (14)	0.0210 (15)	−0.0009 (16)
C20	0.0596 (16)	0.0453 (15)	0.0739 (19)	0.0067 (12)	0.0145 (14)	−0.0015 (13)
C12	0.0742 (19)	0.0606 (18)	0.0526 (16)	−0.0057 (15)	0.0001 (14)	0.0008 (14)
O1A	0.105 (6)	0.081 (5)	0.058 (3)	−0.032 (4)	−0.015 (4)	0.001 (3)
O1B	0.127 (6)	0.074 (4)	0.058 (3)	−0.012 (4)	0.006 (4)	−0.013 (3)
O2A	0.077 (5)	0.059 (3)	0.048 (3)	−0.001 (3)	−0.001 (3)	0.005 (2)
O2B	0.098 (8)	0.091 (5)	0.048 (4)	−0.031 (5)	0.001 (5)	0.011 (5)
C13A	0.094 (6)	0.079 (6)	0.048 (3)	−0.003 (4)	0.000 (5)	0.009 (4)
C13B	0.098 (7)	0.085 (6)	0.049 (4)	−0.012 (5)	0.000 (5)	0.011 (5)
C14A	0.138 (10)	0.121 (9)	0.067 (5)	−0.043 (7)	0.002 (7)	0.016 (6)
C14B	0.104 (8)	0.086 (6)	0.063 (5)	−0.008 (5)	0.008 (5)	0.017 (4)

### Ethyl 2-amino-4-(4-chlorophenyl)-5,6,7,8,9,10-hexahydrocycloocta[b]pyridine-3-carboxylate Geometric parameters (Å, º)

**Table:**

N1—C10	1.336 (3)	C10—C11	1.423 (4)
N1—C9	1.337 (3)	C11—C12	1.478 (4)
N2—C10	1.355 (3)	C15—C16	1.383 (4)
N2—H2A	0.8600	C15—C20	1.383 (4)
N2—H2B	0.8600	C16—C17	1.378 (4)
Cl1—C18	1.744 (3)	C16—H16	0.9300
C1—C2	1.404 (3)	C17—C18	1.367 (4)
C1—C11	1.405 (4)	C17—H17	0.9300
C1—C15	1.498 (4)	C18—C19	1.366 (4)
C2—C9	1.397 (4)	C19—C20	1.383 (4)
C2—C3	1.514 (4)	C19—H19	0.9300
C3—C4	1.518 (4)	C20—H20	0.9300
C3—H3A	0.9700	C12—O1A	1.228 (6)
C3—H3B	0.9700	C12—O2A	1.319 (9)
C4—C5	1.517 (5)	O2A—C13A	1.48 (3)
C4—H4A	0.9700	O2B—C13B	1.41 (3)
C4—H4B	0.9700	C13A—C14A	1.48 (3)
C6—C5	1.486 (5)	C13A—H13A	0.9700
C6—C7	1.558 (6)	C13A—H13B	0.9700
C6—H6A	0.9700	C13B—C14B	1.59 (3)
C6—H6B	0.9700	C13B—H13C	0.9700
C8—C9	1.505 (4)	C13B—H13D	0.9700
C8—C7	1.522 (5)	C14A—H14A	0.9600
C8—H8A	0.9700	C14A—H14B	0.9600
C8—H8B	0.9700	C14A—H14C	0.9600
C7—H7A	0.9700	C14B—H14D	0.9600
C7—H7B	0.9700	C14B—H14E	0.9600
C5—H5A	0.9700	C14B—H14F	0.9600
C5—H5B	0.9700		
			
C10—N1—C9	119.7 (2)	N2—C10—C11	123.0 (2)
C10—N2—H2A	120.0	C1—C11—C10	117.5 (2)
C10—N2—H2B	120.0	C1—C11—C12	124.0 (2)
H2A—N2—H2B	120.0	C10—C11—C12	118.4 (2)
C2—C1—C11	120.1 (2)	C16—C15—C20	118.3 (3)
C2—C1—C15	118.1 (2)	C16—C15—C1	121.5 (2)
C11—C1—C15	121.7 (2)	C20—C15—C1	120.2 (2)
C9—C2—C1	117.3 (2)	C17—C16—C15	121.2 (3)
C9—C2—C3	121.1 (2)	C17—C16—H16	119.4
C1—C2—C3	121.6 (2)	C15—C16—H16	119.4
C2—C3—C4	116.5 (3)	C18—C17—C16	119.4 (3)
C2—C3—H3A	108.2	C18—C17—H17	120.3
C4—C3—H3A	108.2	C16—C17—H17	120.3
C2—C3—H3B	108.2	C19—C18—C17	120.8 (3)
C4—C3—H3B	108.2	C19—C18—Cl1	119.7 (2)
H3A—C3—H3B	107.3	C17—C18—Cl1	119.5 (2)
C5—C4—C3	118.0 (3)	C18—C19—C20	119.7 (3)
C5—C4—H4A	107.8	C18—C19—H19	120.1
C3—C4—H4A	107.8	C20—C19—H19	120.1
C5—C4—H4B	107.8	C19—C20—C15	120.6 (3)
C3—C4—H4B	107.8	C19—C20—H20	119.7
H4A—C4—H4B	107.1	C15—C20—H20	119.7
C5—C6—C7	115.5 (3)	O1A—C12—O2A	117.1 (10)
C5—C6—H6A	108.4	O1A—C12—C11	123.1 (5)
C7—C6—H6A	108.4	O2A—C12—C11	113.1 (10)
C5—C6—H6B	108.4	C12—O2A—C13A	117.5 (15)
C7—C6—H6B	108.4	C14A—C13A—O2A	110.4 (17)
H6A—C6—H6B	107.5	C14A—C13A—H13A	109.6
C9—C8—C7	112.7 (3)	O2A—C13A—H13A	109.6
C9—C8—H8A	109.0	C14A—C13A—H13B	109.6
C7—C8—H8A	109.0	O2A—C13A—H13B	109.6
C9—C8—H8B	109.0	H13A—C13A—H13B	108.1
C7—C8—H8B	109.0	O2B—C13B—C14B	103.6 (17)
H8A—C8—H8B	107.8	O2B—C13B—H13C	111.0
C8—C7—C6	115.9 (3)	C14B—C13B—H13C	111.0
C8—C7—H7A	108.3	O2B—C13B—H13D	111.0
C6—C7—H7A	108.3	C14B—C13B—H13D	111.0
C8—C7—H7B	108.3	H13C—C13B—H13D	109.0
C6—C7—H7B	108.3	C13A—C14A—H14A	109.5
H7A—C7—H7B	107.4	C13A—C14A—H14B	109.5
C6—C5—C4	116.4 (3)	H14A—C14A—H14B	109.5
C6—C5—H5A	108.2	C13A—C14A—H14C	109.5
C4—C5—H5A	108.2	H14A—C14A—H14C	109.5
C6—C5—H5B	108.2	H14B—C14A—H14C	109.5
C4—C5—H5B	108.2	C13B—C14B—H14D	109.5
H5A—C5—H5B	107.3	C13B—C14B—H14E	109.5
N1—C9—C2	123.5 (2)	H14D—C14B—H14E	109.5
N1—C9—C8	114.7 (2)	C13B—C14B—H14F	109.5
C2—C9—C8	121.7 (2)	H14D—C14B—H14F	109.5
N1—C10—N2	115.1 (2)	H14E—C14B—H14F	109.5
N1—C10—C11	121.9 (2)		
			
C11—C1—C2—C9	2.2 (4)	N1—C10—C11—C1	2.0 (4)
C15—C1—C2—C9	−175.5 (2)	N2—C10—C11—C1	−178.5 (2)
C11—C1—C2—C3	−179.0 (2)	N1—C10—C11—C12	−179.0 (3)
C15—C1—C2—C3	3.2 (4)	N2—C10—C11—C12	0.5 (4)
C9—C2—C3—C4	84.5 (3)	C2—C1—C15—C16	−81.3 (3)
C1—C2—C3—C4	−94.3 (3)	C11—C1—C15—C16	101.0 (3)
C2—C3—C4—C5	−74.1 (4)	C2—C1—C15—C20	96.2 (3)
C9—C8—C7—C6	51.0 (4)	C11—C1—C15—C20	−81.5 (3)
C5—C6—C7—C8	56.5 (4)	C20—C15—C16—C17	0.3 (4)
C7—C6—C5—C4	−98.5 (4)	C1—C15—C16—C17	177.8 (3)
C3—C4—C5—C6	66.4 (5)	C15—C16—C17—C18	0.1 (5)
C10—N1—C9—C2	−1.5 (4)	C16—C17—C18—C19	0.1 (4)
C10—N1—C9—C8	−177.5 (2)	C16—C17—C18—Cl1	−178.9 (2)
C1—C2—C9—N1	0.2 (4)	C17—C18—C19—C20	−0.6 (5)
C3—C2—C9—N1	−178.6 (2)	Cl1—C18—C19—C20	178.4 (2)
C1—C2—C9—C8	175.9 (2)	C18—C19—C20—C15	1.0 (5)
C3—C2—C9—C8	−2.8 (4)	C16—C15—C20—C19	−0.9 (4)
C7—C8—C9—N1	86.3 (3)	C1—C15—C20—C19	−178.4 (3)
C7—C8—C9—C2	−89.8 (3)	C1—C11—C12—O1A	−173.8 (10)
C9—N1—C10—N2	−179.2 (2)	C10—C11—C12—O1A	7.3 (10)
C9—N1—C10—C11	0.4 (4)	C1—C11—C12—O2A	−23.5 (8)
C2—C1—C11—C10	−3.3 (4)	C10—C11—C12—O2A	157.6 (8)
C15—C1—C11—C10	174.4 (2)	O1A—C12—O2A—C13A	−31.6 (17)
C2—C1—C11—C12	177.8 (3)	C11—C12—O2A—C13A	176.1 (10)
C15—C1—C11—C12	−4.5 (4)	C12—O2A—C13A—C14A	−145 (2)

### Ethyl 2-amino-4-(4-chlorophenyl)-5,6,7,8,9,10-hexahydrocycloocta[b]pyridine-3-carboxylate Hydrogen-bond geometry (Å, º)

*Cg* is the centroid of pyridine ring (C1/C2/C9/N1/C10/C11).

**Table:**

*D*—H···*A*	*D*—H	H···*A*	*D*···*A*	*D*—H···*A*
N2—H2*B*···O1*A*	0.86	1.96	2.605 (10)	131
N2—H2*A*···N1^i^	0.86	2.29	3.129 (3)	165
C17—H17···*Cg*^ii^	0.93	2.84	3.657 (3)	147

Symmetry codes: (i) −*x*, −*y*−1, −*z*+1; (ii) *x*, *y*+1, *z*.
